# Supplementary material for: Taking the chance!–Interindividual differences in rule-breaking
Source: PLoS One. 2022 Oct 7;17(10):e0274837. doi: 10.1371/journal.pone.0274837 (PMC9544015; doi:10.1371/journal.pone.0274837)
Supplement: S1 File — (DOCX) [file pone.0274837.s001.docx]

**Taking the chance! – Interindividual differences in rule-breaking**

**Highlights**

- Individuals who tend to break the rules to obtain higher payoffs experience significantly more cognitive conflict, measured via response times and mouse movement trajectories, than those who follow the rules.
- Cognitive conflict is more pronounced when violating the rules than when following them.
- Cognitive conflict is more intense in the action planning of rule-breaking than in its execution.
- The Decision-Implementation-Mandatory switch-Inhibition model applies to the cognitive schema of interindividual differences in rule-breaking.
- Personality traits seem to play a role in appreciating behaviours and cognitive characteristics of rule-followers and rule-breakers.
