# Supplementary material for: Taking the chance!–Interindividual differences in rule-breaking
Source: PLoS One. 2022 Oct 7;17(10):e0274837. doi: 10.1371/journal.pone.0274837 (PMC9544015; doi:10.1371/journal.pone.0274837)
Supplement: S2 File — (DOCX) [file pone.0274837.s002.docx]

**Table of contents: Supplementary Material**

1. Methods
   1. Exclusion Data
   2. List of combinations of Stock used per subject in each of the block
   3. Questionnaires
   4. Mouse trajectory parameters
2. Results
   1. Results remain stable using different percentages
   2. Decision-making in the “rule-free” and “rule” part
   3. Post hoc analyses assuming independence of the groups (rule-followers and rule-breakers) when comparing trials associated a different type of consequences.
   4. The influence of frequency. recency. and latency of rule-breaking on reaction times and mouse trajectory parameters
   5. Results of t-tests comparing rule-breaking and rule-following trials in those trials associated to negative consequences
   6. Results regarding the current and last trials responses in rule-breakers
   7. Correlation tables

**Methods**

***Exclusion Data***

Because our study involved different types of responses (e.g., opt for the highest number of stocks during the rule-free part. commit rule-following. commit rule-breaking) within one *rule-breaking task* it was hard to exclude trials by reaction times. Moreover. we are profoundly interested in all of these kinds of responses. Therefore. we opted by limiting the reaction times from 250ms to 5000ms to avoid confounding the data by lack of attention towards the task.

Next, we performed outlier analyses of the reaction times only on the "rule" part of the task because our control analyses revealed that participants in general took more time in the "rule-free" part than in the "rule" part. Because the "rule-free" occurs earlier, the overall slower times clearly exhibited a learning process. Furthermore. the main analyses of this study focused in the "rule" part of the task. As our design includes a between subject factor (rule-followers vs. rule-breakers).

* We are open to share data and scripts with reviewers and readers.

***List of combinations of Stock used per subject in each of the block.***

| **Trial Number** | **Blue**  **Box** | **Orange**  **Box** |
| --- | --- | --- |
| 1 | -5000 | 0 |
| 2 | -3000 | -1000 |
| 3 | -3000 | -500 |
| 4 | -3000 | 500 |
| 5 | -3000 | 0 |
| 6 | -1000 | -3000 |
| 7 | -500 | -3000 |
| 8 | -1000 | -1000 |
| 9 | -1000 | -500 |
| 10 | -1000 | 500 |
| 11 | -1000 | 0 |
| 12 | -500 | -1000 |
| 13 | 0 | -5000 |
| 14 | 0 | -3000 |
| 15 | 0 | 0 |
| 16 | -500 | 500 |
| 17 | -500 | 500 |
| 18 | -500 | 0 |
| 19 | 0 | -1000 |
| 20 | 500 | -3000 |
| 21 | 500 | -1000 |
| 22 | 1000 | -3000 |
| 23 | 1000 | 1000 |
| 24 | 0 | 500 |
| 25 | 0 | -500 |
| 26 | 1000 | -1000 |
| 27 | 3000 | -3000 |
| 28 | 3000 | -1000 |
| 29 | -3000 | 1000 |
| 30 | -3000 | 3000 |
| 31 | -1000 | 1000 |
| 32 | -3000 | -3000 |
| 33 | 500 | 0 |
| 34 | 500 | -500 |
| 35 | 500 | -500 |
| 36 | -1000 | 3000 |
| 37 | -500 | 1000 |
| 38 | -500 | 3000 |
| 39 | 0 | 1000 |
| 40 | -500 | -500 |
| 41 | 1000 | 0 |
| 42 | 1000 | -500 |
| 43 | 1000 | 500 |
| 44 | 0 | 3000 |
| 45 | 0 | 5000 |
| 46 | 500 | 1000 |
| 47 | 500 | 500 |
| 48 | 3000 | 0 |
| 49 | 3000 | -500 |
| 50 | 3000 | 500 |
| 51 | 3000 | 1000 |
| 52 | 500 | 3000 |
| 53 | 1000 | 3000 |
| 54 | 3000 | 3000 |
| 55 | 5000 | 0 |

24 Trials: Positive Consequences

24 Trials: Negative Consequences

7 Trials: Neutral Consequences

***Questionnaires***

Narcissism (13 items)

We evaluated and scored narcissism using the Narcissistic Personality Inventory (13 items; Gentile et al.. 2013).

Maximum possible value was 13. minimum possible value was 0.

Literature:

Cronbach’s Alpha: .73

Validity: .32

Current Study:

Cronbach’s Alpha: .62

Narcissism: grandiose (5 items)

Maximum possible value was 5. minimum possible value was 0.

Literature:

Cronbach’s Alpha: 0.65

Validity: 0.26

Current Study:

Cronbach’s Alpha:.45

Narcissism: leadership (4 items)

Maximum possible value was 4. minimum possible value was 0.

Literature:

Cronbach’s Alpha: 0.66

Validity: 0.32

Current Study:

Cronbach’s Alpha:.54

Narcissism: entitlement (4 items)

Maximum possible value was 4. minimum possible value was 0.

Literature

Cronbach’s Alpha: 0.51

Validity: 0.21

Current Study:

Cronbach’s Alpha:.35

*Risk propensity*

We evaluated and scored risk propensity using the risk propensity questionnaire (2 items; Antoncic et al., 2016). The maximum possible value was 5. minimum possible value was 1.

Literature:

Cronbach’s Alpha: 0.747

Current Study:

Cronbach’s Alpha:.68

*Impulsiveness: Behavioral inhibition and activation systems*

We evaluated and scored Behavioral inhibition and activation systems using the BIS/BAS inventory (24 items; Carver & White. 1994). The scales behavioral inhibition (7 items). behavioral activation drive (4 items). behavioral activation fun seeking (4 items). behavioral activation reward/responsiveness (4 items) had the maximum possible value of 4. minimum possible value of 1. IMPORTANTLY: lower values here indicate high behavioural inhibition/behavioural activation and greater values here indicate low behavioural inhibition/behavioural activation.

Literature:

Cronbach’s Alpha: Reliability: 0.72

Validity: p <0.001

Current Study:

Cronbach’s Alpha: .7

*Big Five personality*

We evaluated and scored Big-Five personality traits using the Big Five inventory (10 items; Rammstedt & John. 2007). Each personality trait (Agreeableness. Conscientiousness. Neuroticism. Openness) was evaluated with two items. The maximum possible value was 5. minimum possible value was 1.

Literature:

Cronbach’s Alpha: 0.75

Validity: 0.11

Current Study:

Cronbach’s Alpha:.45

***Figure S1.*** Stimuli Location on the screen

***Figure S2.*** Additional diagram about the block structure

***1.4 Mouse trajectory parameters***

*Calculation:*

*Maximum absolute distance (MAD):*

1. For every measurement, a straight line is taken from the starting point to the target as a reference line.
2. Then the movement path of participant is taken and broken down into 100 step points.
3. MAD was then calculated as the maximum absolute distance from each of these points to the reference line (distance measured in terms of number of pixels).
4. To calculate MAD, they use this

*d(x) = sqrt( (p1-x)^2 + (p2-y)^2 )*

where, *(x,y)* is the point on reference line and *(p1,p2)* is the point on trajectory line.

1. By squaring and rearranging the above equation they get

*d(x)^2 = (a^2+1)*x^2 + (2*a*b - 2*p1 - 2*p2*a)*x + p1^2 + p2^2 - 2*p2*b + b^2*

1. Now taking the derivative and equating it to zero, we get the value of x

*x0 = (p1 + p2*a - a*b) / (a^2+1)*

1. Then y is calculated

*y0 = a*x0 + b*

where, a and b are the slope and intercept of the reference line.

1. Finally, ‘ad’ is computed as the simple Euclidean distance between the two points P(px,py) and G(x0,y0):

*ad = sqrt((px-x0).^2 + (py-y0).^2)*

1. Then direction is calculated. Deviations towards the opposing target area were coded as positive values and deviations towards the nearest edge of the screen produced negative values.

*direction = sign(py - (a*px+b))*

(Jusyte et. al., 2017, pp. 939–946; Wirth et. al., 2020, pp. 2394–2416)

*In Matlab:*

*function [ad, direction, linecoords] = comp_ad(px,py,a,b)*

This function computes the absolute (Euclidean) distance of a point P (with the 2D-coordinates px and py) and a line, defined by slope a and intercept b. The arguments px and py may be scalars or vectors of the same length. In the latter case, ad will be a vector of distances. Additional output arguments are direction (1 = above the line, 0 = on the line, -1 = below the line) and line coordinates (=linecoords) (structure with the fields x and y for x0 and y0).

*Area under the curve (AUC):*

1. Measured in px^2^.
2. Area between the actual movement and the perfect line.
3. Deviations towards the opposing target area were coded as positive values and deviations towards the nearest edge of the screen produced negative values.
4. AUCs are computed by dividing the area in triangular and square-shaped pieces and adding them up across the trajectory. The output is a vector representing the cumulative AUC across course of the trajectory. (Jusyte et. al., 2017, pp. 939–946; Wirth et. al., 2020, pp. 2394–2416)

*In Matlab:*

*function [ad, direction, linecoords] = comp_ad(px,py,a,b)*

This function computes the absolute (Euclidean) distance of a point P (with the 2D-coordinates px and py) and a line, defined by slope a and intercept b. The arguments px and py may be scalars or vectors of the same length. In the latter case, ad will be a vector of distances. Additional output arguments are direction (1 = above the line, 0 = on the line, -1 = below the line) and line coordinates (=linecoords) (structure with the fields x and y for x0 and y0).

*References*

1. Jusyte, A. et. al., (2017). Smooth criminal: convicted rule-breakers show reduced cognitive conflict during deliberate rule violations (81^st^ ed.). Psychological Research.
2. Wirth, R. et. al., (2020). Design choices: Empirical recommendations for designing two-dimensional finger-tracking experiments (52^nd^ ed.). Behavior Research Methods.

***Results***

**Results remain stable using different percentages**

We have tested and are open to share scripts and data to test different percentages.


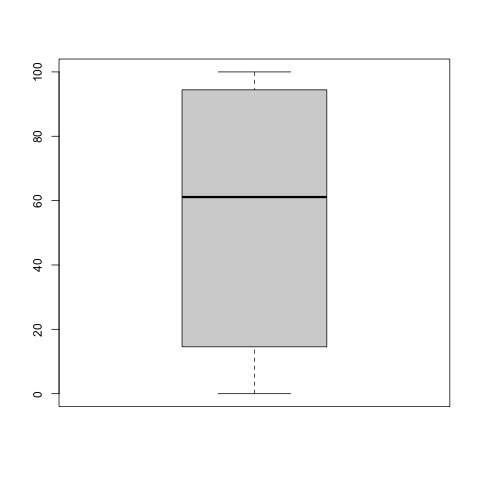

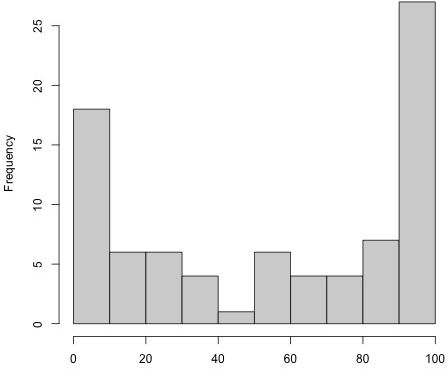


***Figure S3*.** Distribution of the frequency of rule-breaking in trials where rules were violated to obtain benefits in individuals that broke the rule at least once.

In the data reported. the first lower quantile was classified as rule-followers. and the rest as rule-breakers. The results remained the same when trying different percentages ((5%. 10%. 15%. 20%. 55%). We tried 55% as this was the mean of the distribution. However. conceptually we believe the first 25% was a better measurement than the rest of percentages. as a trade-off between losing data and keeping valuable data. What is more. rule-breakers (the rest 75%') of the sample reported explicitly in a questionnaire that they broke a rule in order to get a benefit for themselves.

**Decision-making in the “rule-free” and “rule” part**

Multiple independent mixed 2 x 2 ANOVAs with the experimental part (i.e., “rule” part versus “rule-free” part) as a within group factor and the behavioral tendency (i.e., rule-followers versus rule-breakers) as a between group factor were computed to examine whether there were significant differences on participants behavior (i.e., payoffs. reaction times. mouse trajectory parameters) (see *Figure S3 and Table S1*). The dependent variables are the reaction times, and mouse trajectory parameters.

|  | Value | F | df | df | P | Effect size |
| --- | --- | --- | --- | --- | --- | --- |
| Total time (ms) | 0.541 | 154.400^b^ | 1 | 131 | 0 | 0.541 |
| Initiation time (ms) | 0.404 | 88.936^b^ | 1 | 131 | 0 | 0.404 |
| Movement time (ms) | 0.166 | 26.119^b^ | 1 | 131 | 0 | 0.166 |
| MAD (px) | 0.209 | 34.522^b^ | 1 | 131 | 0 | 0.209 |
| AUC (px^2^) | 0.168 | 26.398^b^ | 1 | 131 | 0 | 0.168 |

***Table S1.*** ANOVA for repeated measurement results

|  |  | Mean Difference | Std. Error | P | 95% CI for B | |
| --- | --- | --- | --- | --- | --- | --- |
|  |  |  |  |  | Lower Bound | Upper Bound |
|  |  |  |  |  |  |  |
| *Total time (ms)* |  |  |  |  |  |  |
| Behavioral tendencies |  |  |  |  |  |  |
| Rule-breakers | Rule-followers | 117.89^*^ | 29.11 | 0 | 60.32 | 175.47 |
|  |  |  |  |  |  |  |
|  |  |  |  |  |  |  |
| Experimental Parts |  |  |  |  |  |  |
| Rule | Rule-free | -197.21^*^ | 11.29 | 0 | -219.55 | -174.87 |
|  |  |  |  |  |  |  |
|  |  |  |  |  |  |  |
|  |  |  |  |  |  |  |
| Behavioral tendency: Experimental Part | |  |  |  |  |  |
| Rule-breakers: Rule | Rule-followers: Rule-free | -79.31 | 31.22 | .07 | -162.31 | 3.68 |
|  | Rule-followers: Rule | 258.21^*^ | 31.22 | 0 | 175.22 | 341.21 |
|  | Rule-breakers: Rule-free | -56.89 | 32.03 | .46 | -142.04 | 28.26 |
| Rule-Breakers: Rule-free | Rule-followers: Rule-free | -22.43 | 31.22 | 1 | -105.42 | 60.57 |
|  | Rule-followers: Rule | 315.1^*^ | 31.22 | 0 | 232.10 | 398.09 |
| Rule-followers: Rule | Rule-followers: Rule-free | -337.53^*^ | 30.39 | 0 | -418.31 | -256.75 |
|  |  |  |  |  |  |  |
| *Initiation time (ms)* |  |  |  |  |  |  |
| Behavioral tendencies |  |  |  |  |  |  |
| Rule-breakers | Rule-followers | 100.64^*^ | 20.05 | 0 | 60.99 | 140.3 |
|  |  |  |  |  |  |  |
|  |  |  |  |  |  |  |
| Experimental Parts |  |  |  |  |  |  |
| Rule | Rule-free | -141.9^*^ | 9.98 | 0 | -161.64 | -122.16 |
|  |  |  |  |  |  |  |
|  |  |  |  |  |  |  |
|  |  |  |  |  |  |  |
| Behavioral tendency: Experimental Part | |  |  |  |  |  |
| Rule-breakers: Rule | Rule-followers: Rule-free | -41.25 | 22.39 | .4 | -100.78 | 18.28 |
|  | Rule-followers: Rule | 194.75^*^ | 22.39 | 0 | 135.22 | 254.27 |
|  | Rule-breakers: Rule-free | -47.79 | 22.97 | .23 | -108.87 | 13.28 |
| Rule-Breakers: Rule-free | Rule-followers: Rule-free | 6.54 | 22.39 | 1 | -52.99 | 66.07 |
|  | Rule-followers: Rule | 242.54^*^ | 22.39 | 0 | 183.01 | 302.07 |
| Rule-followers: Rule | Rule-followers: Rule-free | -236^*^ | 21.79 | 0 | -293.94 | -178.06 |
|  |  |  |  |  |  |  |
| *Movement time (ms)* |  |  |  |  |  |  |
| Behavioral tendencies |  |  |  |  |  |  |
| Rule-breakers | Rule-followers | 17.249 | 27.464 | .53 | -37.08 | 71.58 |
|  |  |  |  |  |  |  |
|  |  |  |  |  |  |  |
| Experimental Parts |  |  |  |  |  |  |
| Rule | Rule-free | -55.311^*^ | 9.043 | 0 | -73.20 | -37.42 |
|  |  |  |  |  |  |  |
|  |  |  |  |  |  |  |
|  |  |  |  |  |  |  |
| Behavioral tendency: Experimental Part | |  |  |  |  |  |
| Rule-breakers: Rule | Rule-followers: Rule-free | -38.06 | 28.92 | 1 | -114.93 | 38.81 |
|  | Rule-followers: Rule | 63.47 | 28.92 | .17 | -13.40 | 140.34 |
|  | Rule-breakers: Rule-free | -9.09 | 29.67 | 1 | -87.96 | 69.77 |
| Rule-Breakers: Rule-free | Rule-followers: Rule-free | -28.97 | 28.92 | 1 | -105.84 | 47.9 |
|  | Rule-followers: Rule | 72.56 | 28.92 | .08 | -4.31 | 149.43 |
| Rule-followers: Rule | Rule-followers: Rule-free | -101.53^*^ | 28.14 | 0 | -176.35 | -26.71 |
|  |  |  |  |  |  |  |

| *Maximum absolute distance (px)* |  |  |  |  |  |  |
| --- | --- | --- | --- | --- | --- | --- |
| Behavioral tendencies |  |  |  |  |  |  |
| Rule-breakers | Rule-followers | 7.36 | 3.84 | .06 | -0.25 | 14.96 |
|  |  |  |  |  |  |  |
|  |  |  |  |  |  |  |
| Experimental Parts |  |  |  |  |  |  |
| Rule | Rule-free | -10.59^*^ | 1.93 | 0 | -14.4 | -6.77 |
|  |  |  |  |  |  |  |
|  |  |  |  |  |  |  |
|  |  |  |  |  |  |  |
| Behavioral tendency: Experimental Part | |  |  |  |  |  |
| Rule-breakers: Rule | Rule-followers: Rule-free | -3.23 | 4.3 | 1 | -14.66 | 8.2 |
|  | Rule-followers: Rule | 18.69^*^ | 4.3 | 0 | 7.26 | 30.12 |
|  | Rule-breakers: Rule-free | 0.74 | 4.41 | 1 | -10.98 | 12.47 |
| Rule-Breakers: Rule-free | Rule-followers: Rule-free | -3.97 | 4.3 | 1 | -15.41 | 7.46 |
|  | Rule-followers: Rule | 17.94^*^ | 4.3 | 0 | 6.51 | 29.38 |
| Rule-followers: Rule | Rule-followers: Rule-free | -21.92^*^ | 4.19 | 0 | -33.04 | -10.79 |
|  |  |  |  |  |  |  |
|  |  |  |  |  |  |  |

| *Area under the curve (px^2^)* |  |  |  |  |  |  |
| --- | --- | --- | --- | --- | --- | --- |
|  |  |  |  |  |  |  |
|  |  | Mean Difference | Std. Error | P | 95% CI for B | |
|  |  |  |  |  | Lower Bound | Upper Bound |
| Behavioral tendencies |  |  |  |  |  |  |
| Rule-breakers | Rule-followers | 1299.22 | 1021.75 | .21 | -722.04 | 3320.49 |
|  |  |  |  |  |  |  |
|  |  |  |  |  |  |  |
| Experimental Parts |  |  |  |  |  |  |
| Rule | Rule-free | -1634.9^*^ | 509.43 | 0 | -2642.68 | -627.14 |
|  |  |  |  |  |  |  |
|  |  |  |  |  |  |  |
|  |  |  |  |  |  |  |
| Behavioral tendency: Experimental Part | |  |  |  |  |  |
| Rule-breakers: Rule | Rule-followers: Rule-free | -335.69 | 1141.71 | 1 | -3370.84 | 2699.47 |
|  | Rule-followers: Rule | 3916.63^*^ | 1141.71 | 0 | 881.47 | 6951.78 |
|  | Rule-breakers: Rule-free | 982.49 | 1171.36 | 1 | -2131.51 | 4096.5 |
| Rule-Breakers: Rule-free | Rule-followers: Rule-free | -1318.18 | 1141.71 | 1 | -4353.34 | 1716.97 |
|  | Rule-followers: Rule | 2934.13 | 1141.71 | .06 | -101.02 | 5969.29 |
| Rule-followers: Rule | Rule-followers: Rule-free | -4252.31^*^ | 1111.25 | 0 | -7206.51 | -1298.11 |

***Table S2.*** Posthoc results after testing significance in ANOVAs.

|  | **Rule** | | | | **Rule-free** | | | |
| --- | --- | --- | --- | --- | --- | --- | --- | --- |
|  | **Rule-breakers** | | **Rule-followers** | | **Rule-breakers** | | **Rule-followers** | |
| Dependent variables | Mean | SD | Mean | SD | Mean | SD | Mean | SD |
| Total time (ms) | 1035.4 | 186.1 | 777.2 | 159.9 | 1092.2 | 150.3 | 1114.7 | 213.9 |
| Initiation time (ms) | 550.8 | 149.2 | 356.1 | 77.7 | 598.6 | 132.6 | 592.1 | 145.9 |
| Movement time (ms) | 484.5 | 189.9 | 421.1 | 130 | 493.6 | 163.3 | 522.6 | 178.9 |
| MAD (px) | 55.7 | 29.9 | 37 | 20.7 | 55 | 21 | 58.9 | 26.5 |
| AUC (px^2^) | 12419.6 | 7770.3 | 8503.0 | 6420.7 | 11437.1 | 4997.9 | 12755.3 | 6796.5 |

***Table S3.*** Descriptives of cognitive related variables

***Figure S4.*** Reaction times and mouse trajectories across experimental parts and interindividual differences of responses towards rules. Yellow indicates

rule-breakers. Pink indicates rule-followers. Significance: * = p < .05.

**Post hoc analyses assuming independence of the groups (rule-followers and rule-breakers) when comparing trials associated a different type of consequences.**

| Behavioral tendency | Type of Consequences | | Mean | Std. Error | *P* | 95% Confidence Interval | |
| --- | --- | --- | --- | --- | --- | --- | --- |
|  |  |  | Difference |  |  | Lower Bound | Upper Bound |
| *Total time (ms)* |  |  |  |  |  |  |  |
| Rule-followers | Positive | Negative | -21.98^*^ | 6.61 | 0 | -38.21 | -5.76 |
|  |  | Neutral | -14.36 | 6.26 | .08 | -29.72 | 1 |
|  | Neutral | Negative | -7.62 | 6.45 | .73 | -23.46 | 8.21 |
| Rule-breakers | Positive | Negative | -128.1^*^ | 14.6 | 0 | -164.02 | -92.19 |
|  |  | Neutral | -73.95^*^ | 8.52 | 0 | -94.91 | -52.98 |
|  | Neutral | Negative | -54.16^*^ | 16.37 | .01 | -94.43 | -13.89 |
|  |  |  |  |  |  |  |  |
| *Initiation time (ms)* |  |  |  |  |  |  |  |
| Rule-followers | Positive | Negative | -10.36^*^ | 3.67 | .02 | -19.36 | -1.37 |
|  |  | Neutral | -2.49 | 3.04 | 1 | -9.93 | 4.96 |
|  | Neutral | Negative | -7.88^*^ | 3.06 | .04 | -15.37 | -0.38 |
| Rule-breakers | Positive | Negative | -52.98^*^ | 8.72 | 0 | -74.44 | -31.53 |
|  |  | Neutral | -36.01^*^ | 5.96 | 0 | -50.67 | -21.36 |
|  | Neutral | Negative | -16.97 | 9.46 | .23 | -40.24 | 6.31 |
|  |  |  |  |  |  |  |  |
| *Movement time (ms)* |  |  |  |  |  |  |  |
| Rule-followers | Positive | Negative | -11.62 | 4.78 | .05 | -23.35 | 0.1 |
|  |  | Neutral | -11.88 | 5.15 | .07 | -24.51 | 0.76 |
|  | Neutral | Negative | 0.26 | 5.63 | 1 | -13.56 | 14.07 |
| Rule-breakers | Positive | Negative | -75.12^*^ | 9.99 | 0 | -99.71 | -50.54 |
|  |  | Neutral | -37.93^*^ | 6.82 | 0 | -54.72 | -21.15 |
|  | Neutral | Negative | -37.19^*^ | 10.71 | 0 | -63.55 | -10.83 |
|  |  |  |  |  |  |  |  |
| *Maximum absolute distance (px)* |  |  |  |  |  |  |  |
| Rule-followers | Positive | Negative | -2.69^*^ | 0.9 | .01 | -4.9 | -0.49 |
|  |  | Neutral | -0.98 | 0.93 | .89 | -3.26 | 1.3 |
|  | Neutral | Negative | -1.71 | 1.25 | .53 | -4.78 | 1.35 |
| Rule-breakers | Positive | Negative | -26.23^*^ | 3.22 | 0 | -34.14 | -18.31 |
|  |  | Neutral | -8.96^*^ | 2.99 | .01 | -16.32 | -1.61 |
|  | Neutral | Negative | -17.26^*^ | 4.34 | 0 | -27.93 | -6.6 |
|  |  |  |  |  |  |  |  |
|  |  |  |  |  |  |  |  |
| *Area under the curve (px^2^)* |  |  |  |  |  |  |  |
| Rule-followers | Positive | Negative | -619.91^*^ | 189.17 | .01 | -1084.07 | -155.74 |
|  |  | Neutral | -274.3 | 322.83 | 1 | -1066.44 | 517.85 |
|  | Neutral | Negative | -345.61 | 342.97 | .95 | -1187.16 | 495.94 |
| Rule-breakers | Positive | Negative | -5696.33^*^ | 709.85 | 0 | -7443.03 | -3949.62 |
|  |  | Neutral | -1502.01 | 686.4 | .10 | -3191.02 | 186.99 |
|  | Neutral | Negative | -4194.31^*^ | 1059.47 | 0 | -6801.33 | -1587.3 |

***Table S4.*** Post hoc analyses assuming independence of the groups (rule-followers and rule-breakers) when comparing trials associated a different type of consequences.

**The influence of frequency. recency. and latency of rule-breaking on reaction times and mouse trajectory parameters**

|  | | | | | | |
| --- | --- | --- | --- | --- | --- | --- |
|  | Coeff. B | Bias | Std.Error | P Value | ***95% CI for B*** | |
|  |  |  |  |  | *Conf. low* | *Conf. high* |
| *Percentage of rule-breaking* |  |  |  |  |  |  |
| Total time (ms) | 2.90 | -.02 | .84 | 0 | 1.23 | 4.6 |
| Initiation time (ms) | 1.98 | .03 | .6 | 0 | .9 | 3.18 |
| Movement time (ms) | .92 | .03 | .82 | .29 | -.54 | 2.59 |
| MAD (px) | .1 | .01 | .15 | .53 | -.17 | .42 |
| AUC (px^2^) | 23.07 | -.03 | 38.69 | .54 | -51.70 | 107.52 |
| *Recency* |  |  |  |  |  |  |
| Total time (ms) | 2.09 | -.03 | .81 | .01 | .5 | 3.65 |
| Initiation time (ms) | 1.53 | .02 | .57 | .01 | .35 | 2.62 |
| Movement time (ms) | 1.53 | -.01 | .56 | .01 | .33 | 2.53 |
| MAD (px) | .01 | .01 | .14 | .97 | -.26 | .29 |
| AUC (px^2^) | 1.06 | .82 | 34.24 | .98 | -64.34 | 68.92 |
| *Latency* |  |  |  |  |  |  |
| Total time (ms) | -4.88 | -.06 | 1.42 | 0 | -8.06 | -2.31 |
| Initiation time (ms) | -3.02 | .02 | 1.09 | .01 | -5.3 | -.87 |
| Movement time (ms) | -1.86 | -.07 | 1.18 | .08 | -4.5 | .11 |
| MAD (px) | -.57 | .02 | .23 | .01 | -.95 | -.1 |
| AUC (px^2^) | -113.76 | 2.82 | 63.54 | .07 | -227.86 | 21.34 |

***Table S5.*** Bootstrap analyses with 1000 permutations of the influence of frequency. recency and latency of rule-breaking on reaction times and mouse trajectory parameters in the "rule" part (N= 63).

|  | *Std.* | *Std.* | *Beta* | *t* | *p* | ***95% CI for B*** | | *R^2^* | *R^2^ adjusted* |
| --- | --- | --- | --- | --- | --- | --- | --- | --- | --- |
|  | *coeff.* | *error* | *coeff.* |  |  | *Conf. low* | *Conf. high* |  |  |
| *Percentage of rule-breaking* |  |  |  |  |  |  |  |  |  |
| Total time (ms) | 2.90 | .91 | .38 | 3.2 | 0 | 1.09 | 4.71 | .14 | .13 |
| Initiation time (ms) | 1.98 | .72 | .33 | 2.76 | .01 | .55 | 3.42 | .11 | .1 |
| Movement time (ms) | .92 | .96 | .12 | .96 | .34 | -1 | 2.83 | .02 | 0 |
| MAD (px) | .1 | .18 | .07 | .55 | .58 | -.26 | .46 | .01 | -.01 |
| AUC (px^2^) | 23.07 | 45.22 | .07 | .51 | .61 | -67.34 | 113.48 | 0 | -.01 |
| *Recency* |  |  |  |  |  |  |  |  |  |
| Total time (ms) | 2.09 | .83 | .31 | 2.51 | .02 | .42 | 3.75 | .09 | .08 |
| Initiation time (ms) | 1.53 | .65 | .29 | 2.35 | .02 | .23 | 2.83 | .08 | .07 |
| Movement time (ms) | 1.53 | .65 | .29 | 2.35 | .02 | .23 | 2.83 | .08 | .07 |
| MAD (px) | .01 | .16 | 0 | .03 | .98 | -.31 | .32 | 0 | -.02 |
| AUC (px^2^) | -.57 | .36 | -.20 | -1.56 | .12 | -1.29 | .16 | .04 | .02 |
| *Latency* |  |  |  |  |  |  |  |  |  |
| Total time (ms) | -4.88 | 1.93 | -.31 | -2.53 | .01 | -8.73 | -1.02 | .1 | .08 |
| Initiation time (ms) | -3.02 | 1.53 | -.25 | -1.98 | .05 | -6.07 | .03 | .06 | .05 |
| Movement time (ms) | -1.86 | 1.98 | -.12 | -.94 | .35 | -5.82 | 2.11 | .01 | 0 |
| MAD (px) | -.57 | .36 | -.20 | -1.56 | .12 | -1.29 | .16 | .04 | .02 |
| AUC (px^2^) | -113.76 | 92.58 | -.16 | -1.23 | .22 | -298.88 | 71.36 | .02 | .01 |

***Table S6.*** The influence of frequency. recency. and latency of rule-breaking on reaction times and mouse trajectory parameters in trials in which there were negative consequences - if following the rule (N= 63).

|  | | | | | | |
| --- | --- | --- | --- | --- | --- | --- |
|  | Coeff. B | Bias | Std.Error | P Value | ***95% CI for B*** | |
|  |  |  |  |  | *Conf. low* | *Conf. high* |
| *Percentage of rule-breaking* |  |  |  |  |  |  |
| Total time (ms) | 2.90 | -.02 | .84 | 0 | 1.23 | 4.6 |
| Initiation time (ms) | 1.98 | .03 | .6 | 0 | .9 | 3.18 |
| Movement time (ms) | .92 | .03 | .82 | .29 | -.54 | 2.59 |
| MAD (px) | .1 | .01 | .15 | .53 | -.17 | .42 |
| AUC (px^2^) | 23.07 | -.03 | 38.69 | .54 | -51.70 | 107.52 |
| *Recency* |  |  |  |  |  |  |
| Total time (ms) | 2.09 | -.03 | .81 | .01 | .5 | 3.65 |
| Initiation time (ms) | 1.53 | .02 | .57 | .01 | .35 | 2.62 |
| Movement time (ms) | 1.53 | -.01 | .56 | .01 | .33 | 2.53 |
| MAD (px) | .01 | .01 | .14 | .97 | -.26 | .29 |
| AUC (px^2^) | 1.06 | .82 | 34.24 | .98 | -64.34 | 68.92 |
| *Latency* |  |  |  |  |  |  |
| Total time (ms) | -4.88 | -.06 | 1.42 | 0 | -8.06 | -2.31 |
| Initiation time (ms) | -3.02 | .02 | 1.09 | .01 | -5.3 | -.87 |
| Movement time (ms) | -1.86 | -.07 | 1.18 | .08 | -4.5 | .11 |
| MAD (px) | -.57 | .02 | .23 | .01 | -.95 | -.1 |
| AUC (px^2^) | -113.76 | 2.82 | 63.54 | .07 | -227.86 | 21.34 |

***Table S7.*** Bootstrap analyses with 1000 permutations of the influence of frequency. recency and latency of rule-breaking on reaction times and mouse trajectory parameters in trials in which there were negative consequences - if following the rule (N= 63).

|  | *Std.*  *coeff.* | *Std.*  *error* | *Beta*  *coeff.* | *t* | *p* | ***95% CI for B*** | | *R^2^* | *R^2^ adjusted* |
| --- | --- | --- | --- | --- | --- | --- | --- | --- | --- |
|  |  |  |  |  |  | *Conf. low* | *Conf. high* |  |  |
| *Percentage of rule-breaking* |  |  |  |  |  |  |  |  |  |
| Total reaction time (ms) | -.629 | .95 | -.083 | -.662 | .511 | -2.527 | 1.27 | .083 | .007 |
| Initiation time (ms) | .482 | .743 | .082 | .649 | .518 | -1.002 | 1.967 | .082 | .007 |
| Movement time (ms) | -1.111 | 1.051 | -.132 | -1.057 | .294 | -3.211 | .989 | .132 | .017 |
| Maximum absolute distance. MAD (px) | -.684 | .276 | -.298 | -2.478 | .016 | -1.236 | -.132 | .298 | -.132 |
| Area under the curve. AUC (px^2^) | -133.451 | 62.732 | -.259 | -2.127 | .037 | -258.811 | -8.092 | .259 | .067 |
| *Percentage of recency of rule-breaking* |  |  |  |  |  |  |  |  |  |
| Total reaction time (ms) | -5.80 | 2.12 | -.034 | -.273 | .785 | -4.815 | 3.656 | .034 | .001 |
| Initiation time (ms) | -1.362 | 1.649 | -.103 | -.826 | .412 | -4.658 | 1.934 | .103 | .011 |
| Movement time (ms) | .782 | 2.356 | .042 | .332 | .741 | -3.926 | 5.491 | .042 | .002 |
| Maximum absolute distance. MAD (px) | -.314 | .642 | -.061 | -.489 | .627 | -1.597 | .969 | .061 | .004 |
| Area under the curve. AUC (px^2^) | -35.599 | 144.382 | -.031 | -.267 | .806 | -324.123 | 252.926 | .031 | .001 |
| *Latency of rule-breaking* |  |  |  |  |  |  |  |  |  |
| Total reaction time (ms) | .793 | 1.406 | .071 | .564 | .575 | -4.815 | .575 | -2.016 | .005 |
| Initiation time (ms) | -.448 | 1.1 | -.051 | -.407 | .685 | -2.647 | 1.751 | .051 | .003 |
| Movement time (ms) | 1.242 | 1.559 | .1 | .796 | .429 | -1.874 | 4.358 | .1 | .01 |
| Maximum Absolute Distance. MAD (px) | .42 | .424 | .124 | .989 | .326 | -.428 | 1.267 | .124 | .015 |
| Area under the curve. AUC (px^2^) | 77.598 | 95.493 | .102 | .813 | .420 | -113.228 | 268.425 | .102 | -.01 |

***Table S8.*** The influence of frequency. recency. and latency of rule-breaking on reaction times and mouse trajectory parameters in trials in which there were negative consequences- if following the rule. and rule violations were committed (rule-breakers. N = 63).

|  | | | | |  |  |  |
| --- | --- | --- | --- | --- | --- | --- | --- |
|  | | Coeff. B | Bias | Std.Error | P Value | ***95% CI for B*** | |
|  |  | |  |  |  | *Conf. low* | *Conf. high* |
| *Percentage of rule-breaking* | | | |  |  |  |  |
| Total time (ms) | -.81 | | .08 | 1.06 | .45 | -2.76 | 1.38 |
| Initiation time (ms) | -.25 | | .04 | .81 | .77 | -1.69 | 1.48 |
| Movement time (ms) | -.55 | | .05 | 1.04 | 0.6 | -2.44 | 1.6 |
| MAD (px) | -.59 | | .01 | .32 | .08 | -1.25 | -.01 |
| AUC (px^2^) | -103.47 | | -.81 | 65.85 | .12 | -237.52 | 23.18 |
| *Recency* |  | |  |  |  |  |  |
| Total time (ms) | -.90 | | .06 | .94 | .34 | -2.64 | 1.01 |
| Initiation time (ms) | -.22 | | -.06 | .74 | .77 | -1.73 | 1.17 |
| Movement time (ms) | -.68 | | .04 | .97 | .49 | -2.43 | 1.28 |
| MAD (px) | -0.6 | | 0 | .25 | .02 | -1.08 | -0.1 |
| AUC (px^2^) | -110.01 | | 3.25 | 51.87 | .05 | -205.30 | -5.52 |
| *Latency* |  | |  |  |  |  |  |
| Total time (ms) | -.07 | | -.13 | 1.61 | .97 | -3.45 | 3.1 |
| Initiation time (ms) | 1.12 | | -.09 | 1.46 | .41 | -1.79 | 3.9 |
| Movement time (ms) | -1.19 | | .04 | 1.51 | .41 | -4.01 | 1.91 |
| MAD (px) | -.34 | | .03 | .38 | .33 | -.96 | .57 |
| AUC (px^2^) | -86.71 | | 3.74 | 76.18 | .19 | -224.63 | 74.96 |

***Table S9.*** Bootstrap analyses with 1000 permutations of the influence of frequency. recency and latency of rule-breaking on reaction times and mouse trajectory parameters in trials in the rule part committed (rule-breakers. N = 63).

**Results of t-tests comparing rule-breaking and rule-following trials in those trials associated to negative consequences**

| Dependent variables | Mean | Std. | Std. Error | 95% Confidence Interval | | t | df | Sig. | Effect size |
| --- | --- | --- | --- | --- | --- | --- | --- | --- | --- |
|  |  | Deviation | Mean | Lower | Upper |  |  |  |  |
| Total time (ms) | -154.53 | 280.87 | 37.20 | -229.05 | -80 | -4.15 | 56 | 0 | -.55 |
| Initiation time (ms) | -97.22 | 166.27 | 22.02 | -141.33 | -53.1 | -4.41 | 56 | 0 | -.59 |
| Movement time (ms) | -97.22 | 166.27 | 22.02 | -141.33 | -53.1 | -4.41 | 56 | 0 | -.28 |
| MAD (px) | -40.78 | 59.85 | 7.93 | -56.67 | -24.9 | -5.15 | 56 | 0 | -.68 |
| AUC (px^2^) | -8654.86 | 9223.37 | 1805.24 | -9223.37 | -5038.53 | -4.79 | 56 | 0 | -.64 |
|  | Mean | Bias | Std. Error | Sig | 95% Confidence Interval | | |  |  |
|  |  |  |  |  | Lower | Upper |  |  |  |
| Total Time (ms) | -154.53 | -.62 | 36.57 | 0 | -224.2 | -85.62 |  |  |  |
| Initiation Time (ms) | -97.22 | -.46 | 22.15 | 0 | -139.08 | -52.83 |  |  |  |
| Movement time (ms) | -57.31 | -1.25 | 26.93 | 0 | -113.05 | -5.35 |  |  |  |
| MAD (px) | -40.78 | -.12 | 7.77 | 0 | -56.67 | -25.56 |  |  |  |
| AUC (px^2^) | -8654.86 | -89.67 | 1783.38 | 0 | -9223.37 | -5372.40 |  |  |  |

***Table S10.*** Results of t-tests comparing rule-breaking and rule-following trials in those trials associated to negative consequences

(rule-breakers. N = 63).

|  | Response to rules | | | |
| --- | --- | --- | --- | --- |
|  | Broken |  | Followed |  |
|  | *Mean* | *SD* | *Mean* | *SD* |
| Total time (ms) | 1183.29 | 223.66 | 1028.76 | 285.69 |
| Initiation time (ms) | 614.57 | 178.72 | 517.35 | 197.62 |
| Movement time (ms) | 568.72 | 238.53 | 511.41 | 208.65 |
| MAD (px) | 90.94 | 57.7 | 50.15 | 35.4 |
| AUC (px^2^) | 11143.24 | 8500.16 | 19798.1 | 9223.37 |

***Table S11.*** Descriptives of t-tests comparing rule-breaking and rule-following trials in those trials associated to negative consequences

(rule-breakers. N = 63).

**Results regarding the current and last trials responses in rule-breakers**

|  |  | Mean Difference | Std. Error | P | 95% CI for B | |
| --- | --- | --- | --- | --- | --- | --- |
|  | |  |  |  | Lower Bound | Upper Bound |
| Current trial: Last trial | |  |  |  |  |  |
| *Total Time (ms)* |  |  |  |  |  |  |
| Broken: Followed | Broken: Broken | 67.51 | 34.75 | 0.32 | -24.92 | 159.94 |
|  | Followed: Broken | -131.14* | 34.75 | 0.00 | -223.57 | -38.71 |
|  | Followed: Followed | -110.17* | 34.75 | 0.01 | -202.60 | -17.74 |
| Followed: Broken | Followed: Followed | 20.97 | 34.75 | 1.00 | -71.46 | 113.39 |
|  | Broken: Broken | 198.65* | 34.75 | <.001 | 106.22 | 291.08 |
| Broken: Broken | Followed: Followed | -177.68* | 34.75 | <.001 | -270.11 | -85.25 |
|  |  |  |  |  |  |  |
| *Initiation time (ms)* |  |  |  |  |  |  |
| Broken: Followed | Broken: Broken | -42.19 | 28.43 | 0.84 | -117.81 | 33.42 |
|  | Followed: Broken | -42.69 | 28.43 | 0.81 | -118.30 | 32.93 |
|  | Followed:Followed | 61.55 | 28.43 | 0.19 | -14.06 | 137.17 |
| Followed: Broken | Followed: Followed | 0.50 | 28.43 | 1.00 | -75.12 | 76.11 |
|  | Broken: Broken | 104.24* | 28.43 | 0.00 | 28.62 | 179.85 |
| Broken: Broken | Followed: Followed | 103.74* | 28.43 | 0.00 | 28.13 | 179.36 |
|  |  |  |  |  |  |  |
| *Movement time (ms)* |  |  |  |  |  |  |

| Broken: Followed | Followed: Followed | 5.96 | 36.81 | 1.00 | -91.95 | 103.86 |
| --- | --- | --- | --- | --- | --- | --- |
|  | Followed: Broken | -88.45 | 36.81 | 0.10 | -186.36 | 9.45 |
|  | Broken: Broken | -67.98 | 36.81 | 0.40 | -165.89 | 29.92 |
| Followed: Broken | Followed: Followed | 94.41 | 36.81 | 0.07 | -3.49 | 192.31 |
|  | Broken: Broken | 20.47 | 36.81 | 1.00 | -77.43 | 118.37 |
| Broken: Broken | Followed: Followed | 73.94 | 36.81 | 0.27 | -23.96 | 171.84 |
|  |  |  |  |  |  |  |
| *Maximum absolute distance (px)* | |  |  |  |  |  |
| Broken: Followed | Followed: Followed | 3.16 | 7.80 | 1.00 | -17.58 | 23.89 |
|  | Followed: Broken | -38.91* | 7.80 | <.001 | -59.64 | -18.17 |
|  | Broken: Broken | -28.68* | 7.80 | 0.00 | -49.42 | -7.95 |
| Followed: Broken | Followed: Followed | 42.06* | 7.80 | <.001 | 21.33 | 62.80 |
|  | Broken: Broken | 10.22 | 7.80 | 1.00 | -10.51 | 30.96 |
| Broken: Broken | Followed: Followed | 31.84* | 7.80 | <.001 | 11.10 | 52.57 |
|  |  |  |  |  |  |  |
| Area under the curve |  |  |  |  |  |  |

| Broken: Followed | Followed: Followed | 728.69 | 1815.06 | 1.00 | -4098.63 | 5556.00 |
| --- | --- | --- | --- | --- | --- | --- |
|  | Followed: Broken | -8272.89* | 1815.06 | <.001 | -13100.20 | -3445.57 |
|  | Broken: Broken | -5632.72* | 1815.06 | 0.01 | -10460.03 | -805.40 |
| Followed: Broken | Followed: Followed | 9001.57* | 1815.06 | <.001 | 4174.26 | 13828.89 |
|  | Broken: Broken | 2640.17 | 1815.06 | 0.88 | -2187.15 | 7467.48 |
| Broken: Broken | Followed: Followed | 6361.40* | 1815.06 | 0.00 | 1534.09 | 11188.72 |

***Table S12.*** *AN*OVA Statistics of the current and last trial responses in rule-breakers during the "rule" part. Significanc = p < .*05.*

|  | Followed: Followed | | Broken: Followed | | Followed: Broken | | Broken: Broken | |
| --- | --- | --- | --- | --- | --- | --- | --- | --- |
|  | Mean | SD | Mean | SD | Mean | SD | Mean | SD |
| Total time (ms) | 980.64 | 184.67 | 1048.15 | 152.19 | 1179.29 | 207.83 | 1158.33 | 227.42 |
| Initiation time (ms) | 519.46 | 146.95 | 581.01 | 139.23 | 623.70 | 178.65 | 623.20 | 170.17 |
| Movement time (ms) | 461.19 | 174.47 | 467.14 | 175.18 | 555.60 | 240.20 | 535.13 | 227.84 |
| MAD (px) | 44.84 | 24.35 | 48.00 | 27.09 | 86.90 | 57.23 | 76.68 | 55.28 |
| AUC (px^2^) | 10004.66 | 6422.46 | 10733.34 | 7114.10 | 19006.23 | 9223.37 | 16366.06 | 9223.37 |

***Table S13.*** Descriptives of the current and last trial responses in rule-breakers

|  |  | Mean Diff. | Std. Error | P | 95% CI for B | |
| --- | --- | --- | --- | --- | --- | --- |
|  |  |  |  |  | Lower Bound | Upper Bound |
|  |  |  |  |  |  |  |
| Current trial: Last trial |  |  |  |  |  |  |
| *Total time (ms)* |  |  |  |  |  |  |
| Broken: Followed | Followed:Followed | 135.15 | 54.03 | 0.08 | -8.69 | 279.00 |
|  | Followed: Broken | -35.17 | 52.96 | 1.00 | -176.16 | 105.82 |
|  | Broken: Broken | -14.11 | 52.96 | 1.00 | -155.10 | 126.88 |
| Followed: Broken | Followed: Followed | 170.32* | 47.88 | 0.00 | 42.84 | 297.80 |
|  | Broken: Broken | 21.06 | 46.67 | 1.00 | -103.20 | 145.31 |
| Broken: Broken | Followed: Followed | 149.26* | 47.88 | 0.01 | 21.78 | 276.75 |
|  |  |  |  |  |  |  |
| *Initiation time (ms)* |  |  |  |  |  |  |
| Broken: Followed | Followed:Followed | 105.08 | 42.03 | 0.08 | -6.82 | 216.98 |
|  | Followed: Broken | -13.20 | 41.20 | 1.00 | -122.89 | 96.48 |
|  | Broken: Broken | -9.93 | 41.20 | 1.00 | -119.61 | 99.75 |
| Followed: Broken | Followed: Followed | 118.28* | 37.25 | 0.01 | 19.11 | 217.46 |
|  | Broken: Broken | 3.27 | 36.31 | 1.00 | -93.39 | 99.94 |
| Broken: Broken | Followed: Followed | 115.01* | 37.25 | 0.01 | 15.84 | 214.19 |
|  |  |  |  |  |  |  |
| *Movement time (ms)* |  |  |  |  |  |  |
| Broken: Followed | Followed: Followed | 30.07 | 46.76 | 1.00 | -94.42 | 154.57 |
|  | Followed: Broken | -21.96 | 45.83 | 1.00 | -143.99 | 100.06 |
|  | Broken: Broken | -4.18 | 45.83 | 1.00 | -126.20 | 117.85 |
| Followed: Broken | Followed: Followed | 52.04 | 41.44 | 1.00 | -58.30 | 162.37 |
|  | Broken: Broken | 17.79 | 40.39 | 1.00 | -89.75 | 125.33 |
| Broken: Broken | Followed: Followed | 34.25 | 41.44 | 1.00 | -76.08 | 144.59 |
|  |  |  |  |  |  |  |

| *Maximum absolute distance* | |  |  |  |  |  |
| --- | --- | --- | --- | --- | --- | --- |
| Broken: Followed | Followed: Followed | 18.16 | 11.15 | 0.63 | -11.54 | 47.85 |
|  | Followed: Broken | -22.68 | 10.93 | 0.24 | -51.79 | 6.43 |
|  | Broken: Broken | -12.98 | 10.93 | 1.00 | -42.08 | 16.13 |
| Followed: Broken | Followed: Followed | 40.84* | 9.88 | <.001 | 14.52 | 67.16 |
|  | Broken: Broken | 9.70 | 9.63 | 1.00 | -15.95 | 35.36 |
| Broken: Broken | Followed: Followed | 31.13* | 9.88 | 0.01 | 4.82 | 57.45 |
|  |  |  |  |  |  |  |
| *Area under the curve* (px^2^) | |  |  |  |  |  |
| Broken: Followed | Followed: Followed | 3941.10 | 2512.81 | 0.71 | -2749.11 | 10631.30 |
|  | Followed: Broken | -4653.05 | 2462.97 | 0.36 | -11210.57 | 1904.46 |
|  | Broken: Broken | -2100.45 | 2462.97 | 1.00 | -8657.96 | 4457.07 |
| Followed: Broken | Followed: Followed | 8594.15* | 2227.02 | <.001 | 2664.84 | 14523.46 |
|  | Broken: Broken | 2552.61 | 2170.63 | 1.00 | -3226.57 | 8331.78 |
| Broken: Broken | Followed: Followed | 6041.54* | 2227.02 | 0.04 | 112.24 | 11970.85 |

***Table S14.*** ANOVA Statistics of the current and last trial responses in rule-breakers in trials associated with negative consequences. The asterisks * mark significant results with a significance level of 0.05.

|  | Followed: Followed | | Broken: Followed | | Followed: Broken | | Broken: Broken | |
| --- | --- | --- | --- | --- | --- | --- | --- | --- |
|  | Mean | SD | Mean | SD | Mean | SD | Mean | SD |
| Total time (ms) | 1011.75 | 279.97 | 1146.90 | 348.07 | 1182.07 | 212.42 | 1161.01 | 224.09 |
| Initiation time (ms) | 508.18 | 194.61 | 613.26 | 282.80 | 626.46 | 181.50 | 623.19 | 170.90 |
| Movement time (ms) | 503.57 | 204.26 | 533.64 | 238.10 | 555.61 | 239.40 | 537.82 | 225.55 |
| MAD (px) | 33.88 | 4.49 | 65.59 | 62.31 | 88.27 | 60.38 | 78.56 | 56.60 |
| AUC (px^2^) | 10677.21 | 8539.21 | 14618.31 | 9223.37 | 19271.36 | 9223.37 | 16718.75 | 9223.37 |

***Table S15*.** Descriptives of the current and last trial responses in rule-breakers in trials associated with negative consequences

|  | Paired Differences | |  |  |  |  |  |  |
| --- | --- | --- | --- | --- | --- | --- | --- | --- |
|  | Mean | Std. Deviation | Std. Error Mean | 95% Confidence Interval of the Difference | | | |  |
|  |  |  |  | Lower | Upper | t | df | Two-Sided p |
| Total time (ms) | 1167.31 | 217.25 | 19.35 | 1129.00 | 1205.61 | 60.31 | 125.00 | <.001 |
| Movement time (ms) | 543.86 | 233.41 | 20.79 | 502.71 | 585.01 | 26.16 | 125.00 | <.001 |
| Initiation time (ms) | 621.95 | 173.76 | 15.48 | 591.31 | 652.59 | 40.18 | 125.00 | <.001 |
| MAD (px) | 80.29 | 56.32 | 5.02 | 70.36 | 90.22 | 16.00 | 125.00 | <.001 |
| AUC (px^2^) | 9223.37 | 9223.37 | 1134.19 | 9223.37 | 9223.37 | 15.59 | 125.00 | <.001 |

***Table S16.*** Paired T-Tests with paired Differences and Significance leve in trials associated with negative consequences when participants broke the rule Comparison between breaking the rule in the current trial after following it versus breaking the rule in the current trial after have recently broken it in the las trial.

|  |  | Standardizer^(a)^ | Point Estimate | 95% Confidence Interval | |
| --- | --- | --- | --- | --- | --- |
|  |  |  |  | Lower | Upper |
| Total time (ms) | Cohen's d | 217.25 | 5.37 | 4.68 | 6.06 |
|  | Hedges' correction | 218.57 | 5.34 | 4.66 | 6.02 |
| Initiation time (ms) | Cohen's d | 233.41 | 2.33 | 1.99 | 2.67 |
|  | Hedges' correction | 234.83 | 2.32 | 1.98 | 2.65 |
| Movement time (ms) | Cohen's d | 173.76 | 3.58 | 3.10 | 4.05 |
|  | Hedges' correction | 174.81 | 3.56 | 3.08 | 4.03 |
| Maximum absolute distance (px) | Cohen's d | 56.32 | 1.43 | 1.18 | 1.67 |
|  | Hedges' correction | 56.66 | 1.42 | 1.17 | 1.66 |
| Area under the curve (px^2^) | Cohen's d | 9223.37 | 1.39 | 1.14 | 1.63 |
|  | Hedges' correction | 9223.37 | 1.38 | 1.14 | 1.62 |

***Table S17.*** *Paired Samples Effect Sizes corresponding to talbe S16. ^(a)^The denominator used in estimating the effect sizes. Cohen's d uses the sample standard deviation of the mean difference. Hedges' correction uses the sample standard deviation of the mean difference. plus a correction factor.*

|  | Followed: Broken | | Broken: Broken | |
| --- | --- | --- | --- | --- |
|  | Mean | SD | Mean | SD |
| Total time (ms) | 1179.29 | 207.83 | 1158.33 | 227.42 |
| Initiation time (ms) | 623.70 | 178.65 | 623.20 | 170.17 |
| Movement time (ms) | 555.60 | 240.20 | 535.13 | 227.84 |
| MAD (px) | 86.90 | 57.23 | 76.68 | 55.28 |
| AUC (px^2^) | 19006.23 | 9223.37 | 16366.06 | 9223.37 |

***Table S18.*** Descriptives of the current and last trial responses in rule-breakers in trials associated with negative consequences when participants broke the rule Comparison between breaking the rule in the current trial after following it versus breaking the rule in the current trial after have recently broken it in the las trial.

**Correlation tables**

**Table 3. Correlation table of rule-followers versus rule-breakers and personality, and across individuals in these two groups during the "rule" part**

| **Variables** | **1** | **2** | **3** | **4** | **5** | **6** | **7** | **8** | **9** |
| --- | --- | --- | --- | --- | --- | --- | --- | --- | --- |
| Rule-followers versus rule-breakers ^b^ |  | -.06 | .06 | -.09 | -.05 | -.03 | .05 | .09 | .02 |
| *Rule-followers* |  |  |  |  |  |  |  |  |  |
| Total pay-off |  | -.1 | -.05 | -.04 | -0.13 | 0.08 | 0.04 | -0.03 | -0.08 |
| Total time (ms) |  | -0.13 | .12 | -0.12 | -0.01 | -0.04 | .31* | 0.2 | 0.09 |
| Initiation time (ms) |  | 0.08 | .06 | 0.14 | 0.16 | -0.04 | 0.22 | 0.2 | 0.1 |
| Movement time (ms) |  | 0.11 | .1 | -.24* | -0.11 | -0.03 | .25* | 0.13 | 0.05 |
| AUC (px^2^) |  | 0.08 | .11 | -.04 | -0.14 | -0.02 | -0.17 | -0.17 | -0.15 |
| MAD (px) |  | -0.05 | .12 | -.07 | -0.16 | 0.01 | -0.13 | -0.14 | -0.15 |
| *Rule-breakers* |  |  |  |  |  |  |  |  |  |
| Total pay-off |  | -0.36 | -.21* | -0.2 | -.21* | 0.19 | .28* | .29* | 0.24 |
| Total time (ms) |  | -.36* | -0.21 | -0.15 | -0.2 | -0.17 | .27* | .26* | 0.18 |
| Initiation time (ms) |  | 0 | 0.07 | 0.16 | 0.13 | 0.02 | -0.19 | -0.16 | -0.08 |
| Movement time (ms) |  | -.38* | -0.19 | -0.13 | -0.16 | -0.14 | .25* | .24* | 0.2 |
| AUC (px^2^) |  | -0.16 | 0.2 | 0.06 | -0.24 | -0.16 | -0.1 | 0.14 | 0.01 |
| MAD (px) |  | -0.05 | -0.04 | -0.02 | -0.01 | -.26* | 0.04 | 0.07 | -0.01 |

**Note:** 1 Grandiose narcissism, 2 Agreeableness, 3 Conscientiousness, 4 Extraversion, 5 Risk propensity, 6 BAS drive, 7 BAS fun seeking, 8 BAS reward, 9 BIS. * = p value is less than .05, AUC = area under the curve, MAD = Maximum absolute distance, ^b^ 1 = rule-followers. 2 = rule-breakers. Further correlation analyses can be found in the supplementary material (Tables S19-S23).

| **Variables** | **1** | **2** | **3** | **4** | **5** | **6** | **7** | **8** | **9** | **10** | **11** | **12** | **13** |
| --- | --- | --- | --- | --- | --- | --- | --- | --- | --- | --- | --- | --- | --- |
| Total pay-off | -0.05 | -0.15 | -.19* | 0.11 | -0.13 | 0.059 | 0.187 | -0.005 | 0.122 | 0.08 | 0.15 | 0.14 | -0.06 |
| Total time (ms) | .24* | -0.08 | -0.11 | 0.1 | -0.13 | 0.055 | 0.145 | 0.022 | 0.111 | -0.08 | .21* | 0.15 | -0.03 |
| Initiation time (ms) | -0.1 | -0.04 | -0.1 | 0.11 | 0.01 | 0.04 | 0.06 | -0.026 | 0.039 | -0.01 | 0.13 | 0.11 | -0.09 |
| Movement time (ms) | -0.014 | -0.07 | -0.05 | 0.04 | -.18* | 0.027 | 0.08 | 0.031 | 0.068 | -0.1 | 0.16 | 0.09 | 0.05 |
| AUC (px^2^) | 0.06 | 0.01 | 0.03 | 0.06 | -.22* | 0.027 | 0.004 | -0.063 | -0.013 | -0.02 | 0.04 | -0.01 | -0.06 |
| MAD (px) | 0.08 | 0.02 | 0.03 | 0.04 | -.22* | 0.088 | 0.135 | 0.087 | 0.153 | -0.04 | 0.04 | -0.02 | -0.05 |

***Table S19.*** Correlation of personality across all variables in rule-followers and rule-breakers during the "rule" part

1 Conscientiousness, 2 Extraversion, 3 Agreeableness, 4 Neuroticism, 5 Risk propensity, 6 Narcissism, 7 Narcissism: leadership, 8 Narcissism: grandiose, 9 Narcissism: entitlement, 10 BAS drive, 11 BAS fun seeking, 12 BAS reward responsiveness, 13 BIS. Correlation significant at the .05 level (2 – tailed) represented with asterisk (*).

BIS: Behavioural inhibition system. BAS: Behavioural activation system. No correlation was found to be significant between rule-followers versus rule-breakers, which was reported in the manuscript.

Descriptives

| **Variables** | **Mean (min/max)** | **SD** |
| --- | --- | --- |
| 1. Age | 25.2 (18/68) | 7.1 |
| 2. Sex ^a^ | NA | 0.5 |
|  |  |  |
|  |  |  |
| 4. Total pay-off | 250883.4 (115000/342000) | 57955.9 |
| 5. Total time (ms) | 934.7 (500/1602) | 248.3 |
| 6. Initiation time (ms) | 463.2 (173.2/1131) | 170.5 |
| 7. Movement time (ms) | 471.6 (219.6/1246) | 183.7 |
| 8. Area under the curve (px^2^) | 7164.7 (-6076/58434.8) | 10656.4 |
| 9. Maximum absolute distance (px) | 29.2 (-26.6/205.6) | 42.3 |
| 11. Conscientiousness | 3.2 (1/5) | 0.8 |
| 12. Extraversion | 3.4 (1.5/5) | 0.9 |
| 13. Agreeableness | 3.5 (1/5) | 0.8 |
| 14. Neuroticism | 2.9 (1/5) | 0.9 |
| 15. Risk propensity | 3.2 (1/5) | 0.9 |
| 16. Narcissism | 12.8(8/20) | 2.6 |
| 17. Narcissism: leadership | 9 (4/15) | 2.3 |
| 18. Narcissism: grandiose | 4.6 (3/7) | 1.3 |
| 19. Narcissism: entitlement | 2.7 (1/6) | 1.3 |
| 20. BAS drive | 5.4 (4/8) | 1.2 |
| 21. BAS fun seeking | 8.3 (4/14) | 2.1 |
| 22. BAS reward responsiveness | 7.6 (4/11) | 1.6 |
| 23. BIS | 14.9 (7/23) | 3.5 |

| **Variables** | **1** | **2** | **3** | **4** | **5** | **6** | **7** | **8** | **9** | **10** | **11** | **12** | **13** | **14** |
| --- | --- | --- | --- | --- | --- | --- | --- | --- | --- | --- | --- | --- | --- | --- |
| Total pay-off | -0.13 | -0.1 | -0.21 | -0.04 | -0.11 | -0.02 | -0.08 | 0.08 | 0.04 | -0.03 | -0.058 | -0.077 | 0.029 | -0.054 |
| Total time (ms) | -0.01 | 0 | 0 | -0.12 | 0.11 | 0.11 | 0.09 | -0.04 | .31* | 0.2 | -0.058 | -0.116 | 0.183 | 0.001 |
| Initiation time (ms) | 0.16 | -0.13 | 0.15 | 0.14 | -0.03 | -0.09 | 0.1 | -0.04 | 0.22 | 0.2 | -0.159 | -0.178 | -0.116 | -0.228 |
| Movement time (ms) | -0.11 | 0.08 | -0.08 | -.24* | 0.16 | 0.19 | 0.05 | -0.03 | .25* | 0.13 | -0.043 | -0.141 | -0.052 | -0.115 |
| MAD (px) | -0.14 | 0.11 | 0 | -0.04 | 0.16 | 0.14 | -0.15 | -0.02 | -0.17 | -0.17 | -0.17 | -0.135 | -0.112 | -0.212 |
| AUC (px^2^) | -0.16 | 0.08 | -0.02 | -0.07 | 0.15 | 0.15 | -0.15 | 0.01 | -0.13 | -0.14 | 0.121 | 0.134 | 0.214 | 0.232 |

***Table S20.*** Correlation of personality across all variables in rule-followers during the "rule" part

Correlation significant at the .05 level (2 – tailed) represented with asterisk (*).

BIS: Behavioural inhibition system. BAS: Behavioural activation system

1 Risk propensity, 2 Agreeableness, 3 Conscientiousness, 4 Extraversion, 5 Openness, 6 Neuroticism, 7 BIS, 8 BAS drive, 9 BAS fun seeking, 10 BAS reward responsiveness, 11 Narcissism: leadership, 12 Narcissism: grandiose, 13 Narcissism: entitlement, 14 Narcissism

| **Variables** | **Mean (min/max)** | **SD** |  |
| --- | --- | --- | --- |
| 1. Age | 25(18/68) | 6 |  |
| 2. Sex ^a^ | 1.5(1/2) | .5 |  |
| 3. Total pay-off | 250883.4 (115000/342000) | 19131.1 |  |
| 4. Total time (ms) | 934.7 (500/1602) | 159.8 |  |
| 5. Initiation time (ms) | 463.2 (173.2/1131) | 77.7 |  |
| 6. Movement time (ms) | 471.6 (219.6/1246) | 129.9 |  |
| 7. Maximum absolute distance (px) | 29.2 (-26.6/205.6) | 20.6 |  |
| 8. Area under the curve (px^2^) | 7164.7 (-6076/58434.8) | 6420.6 |  |
| 9. Risk propensity | 3.2 (1/5) | 0.9 |  |
| 10. Agreeableness | 3.5 (1/5) | 0.7 |  |
| 11. Conscientiousness | 3.2 (1/5) | 0.7 |  |
| 12. Extraversion | 3.4 (1.5/5) | 0.9 |  |
| 13. Openness | 3.4 (1/5) | 0.7 |  |
| 14. Neuroticism | 2.9 (1/5) | 0.9 |  |
| 15. BIS | 14.9 (7/23) | 3.3 |  |
| 16. BAS drive | 9 (4/15) | 2.1 |  |
| 17. BAS fun seeking | 8.3 (4/14) | 1.9 |  |
|  |  |  |  |
| 18. BAS reward responsiveness | 7.6 (4/11) | 1.7 |  |
| 16. Narcissism | 13.1(8/20) | 7.4 |  |
| 17. Narcissism: leadership | 5.4(4/8) | 1.7 |  |
| 18. Narcissism: grandiose | 2.96(1/6) | 2.1 |  |
| 19. Narcissism: entitlement | 4.7(3/7) | 1.5 |  |
|  |  |  |  |

| **Variables** | **1** | **2** | **3** | **4** | **5** | **6** | **7** | **8** | **9** | **10** | **11** | **12** | **13** | **14** |  |
| --- | --- | --- | --- | --- | --- | --- | --- | --- | --- | --- | --- | --- | --- | --- | --- |
|  |  |  |  |  |  |  |  |  |  |  |  |  |  |  |  |
| Total pay-off | -.21* | -0.36 | -.21* | -0.2 | -0.11 | 0.96 | 0.19 | .28* | .29* | 0.24 | 0.109 | -0.177 | 0.035 | -0.026 |  |
| Frequency of rule-breaking | -0.2 | -.36* | -0.21 | -0.15 | -0.08 | 0.09 | -0.17 | .27* | .26* | 0.18 | 0.04 | 0.003 | -0.014 | 0.019 |  |
| Recency | 0.13 | 0 | 0.07 | 0.16 | -0.03 | 0.05 | 0.02 | -0.19 | -0.16 | -0.08 | 0.171 | 0.015 | 0.051 | 0.112 |  |
| Latency | -0.16 | -.38* | -0.19 | -0.13 | -0.05 | 0.07 | -0.14 | .25* | .24* | 0.2 | 0.002 | -0.015 | 0.102 | 0.038 |  |
| Total time (ms) | -0.24 | -0.16 | 0.2 | 0.06 | 0 | 0 | -0.16 | -0.1 | 0.14 | 0.01 | 0.166 | 0.027 | -0.03 | 0.08 |  |
| Initiation time (ms) | -0.01 | -0.05 | -0.04 | -0.02 | 0.03 | 0.15 | -.26* | 0.04 | 0.07 | -0.01 | 0.036 | 0.041 | 0.011 | 0.044 |  |
| Movement time (ms) | -0.23 | -0.11 | 0.23 | 0.08 | -0.02 | 0.11 | 0.04 | -0.13 | 0.08 | 0.03 | 0.109 | -0.177 | 0.035 | -0.026 |  |
| MAD (px) | -.28* | 0.01 | 0.18 | 0.21 | -0.14 | -0.1 | 0 | -0.04 | 0.15 | 0.04 | 0.048 | 0.003 | -0.014 | 0.019 |  |
| AUC (px^2^) | -.26* | 0.01 | .21* | .12* | -.16* | -.08* | 0 | -0.03 | .16* | 0.06 | 0.171 | 0.015 | 0.051 | 0.112 |  |

***Table S21.*** Correlation of personality across all variables in rule-breakers during the rule-part

Correlation significant at the .05 level (2 – tailed) represented with asterisk (*).

BIS: Behavioural inhibition system. BAS: Behavioural activation system

1 Risk propensity, 2 Agreeableness, 3 Conscientiousness, 4 Extraversion, 5 Openness, 6 Neuroticism, 7 BIS, 8 BAS drive, 9 BAS fun seeking, 10 BAS reward responsiveness, 11 Narcissism: leadership, 12 Narcissism: grandiose, 13 Narcissism: entitlement+

**Descriptives**

| **Variables** | **Mean** | **SD** |
| --- | --- | --- |
|  | **(min/max)** |  |
| 1. Age | 24.8 (18/68) | 6.3 |
| 2. Sex ^a^ | 1.5 (1/2) | 0.5 |
| 3. Total pay-off | 303182.53 (179500/342000) | 41463.6 |
| 4. Frequency of rule-breaking | 22.1 (1.2/38.1) | 12.4 |
| 5. Recency | 69.6 (12.5/100) | 30 |
| 6. Latency | 45.5 (33/101) | 16.1 |
| 7. Total time (ms) | 1088.2 (604.5/1602.2) | 225.1 |
| 8. Initiation time (ms) | 566.1 (241/1131) | 177 |
| 9. Movement time (ms) | 522.0 (223.2/1245.9) | 215.3 |
| 10. Maximum absolute distance (px) | 48.8 (-10.8/205.5) | 45.8 |
| 11. Area under the curve (px^2^) | 11416.9 (2384.2/58434.8) | 11398.8 |
| 12. Risk propensity | 3.1 (1.5/5) | 0.8 |
| 13. Agreeableness | 3.4 (1/5) | 0.9 |
| 14. Conscientiousness | 3.2 (2/5) | 0.7 |
| 15. Extraversion | 3.3 (1.5/5) | 0.9 |
| 16. Openness | 3.4 (1/5) | 0.7 |
| 17. Neuroticism | 2.9 (1/5) | 0.9 |
| 18. BIS | 15 (7/23) | 3.6 |
| 19 BAS drive | 8.8 (5/15) | 2.3 |
| 20. BAS fun seeking | 8.3 (4/14) | 2.1 |
| 21. BAS reward responsiveness | 7.7 (4/11) | 1.5 |
| 16. Narcissism | 13.12(8/20) | 2.7  2.7 |
| 17. Narcissism: leadership | 4.7(3/7) | 1.3 |
| 18. Narcissism: grandiose | 2.9(1/6) | 1.4 |
| 19. Narcissism: entitlement | 5.4(4/8) | 1.2 |

| **Variables** | **1** | **2** | **3** | **4** | **5** | **6** | **7** | **8** | **9** | **10** | **11** | **12** | **13** | **14** |
| --- | --- | --- | --- | --- | --- | --- | --- | --- | --- | --- | --- | --- | --- | --- |
| Total pay-off | -0.21 | -.36* | -0.21 | -0.2 | -0.11 | 0.09 | -0.19 | .28* | .29* | 0.24 | -0.009 | -0.178 | 0.035 | -0.224 |
| Frequency of rule-breaking | -0.2 | -.36* | -0.21 | -0.15 | 0.08 | 0.09 | -0.17 | .27* | .26* | 0.18 | 0.137 | -0.013 | -0.014 | 0.071 |
| Recency | 0.13 | 0 | 0.07 | 0.16 | -0.03 | 0.05 | 0.02 | -0.19 | -0.16 | -0.08 | 0.131 | -0.042 | -0.002 | 0.04 |
| Latency | -0.16 | -.38* | -0.19 | -0.13 | -0.05 | 0.07 | -0.14 | .25* | 0.24 | 0.2 | 0 | -0.047 | -0.029 | -0.113 |
| Total time (ms) | -0.21 | -0.09 | 0.23 | 0.09 | 0 | 0.01 | -0.14 | -0.16 | 0.11 | -0.07 | 0.139 | -0.061 | 0.012 | 0.038 |
| Initiation time (ms) | 0 | -.01* | -0.01 | 0 | 0.01 | 0.14 | -0.26 | 0 | 0.03 | -0.05 | 0.131 | -0.041 | 0.041 | 0.104 |
| Movement time (ms) | 0.22 | 0.08 | .24* | 0.09 | -0.01 | -0.1 | 0.05 | -0.17 | 0.08 | -0.03 | -0.044 | -0.061 | 0.084 | -0.005 |
| AUC (px^2^) | -.25* | 0 | 0.15 | 0.12 | -0.11 | -0.09 | 0 | -0.08 | 0.11 | -0.05 | 0.168 | 0.007 | -0.025 | 0.109 |
| MAD (px) | -.23* | 0 | 0.19 | 0.12 | -0.14 | -0.07 | 0.01 | -0.08 | 0.12 | -0.01 | -0.01 | 0.072 | -0.013 | 0.082 |

***Table S22.*** Correlation of personality across all variables in rule-breakers in trials associated with negative consequences

Correlation significant at the .05 level (2 – tailed) represented with asterisk (*).

BIS: Behavioural inhibition system. BAS: Behavioural activation system

1 Risk propensity, 2 Agreeableness, 3 Conscientiousness, 4 Extraversion, 5 Openness, 6 Neuroticism, 7 BIS, 8 BAS drive, 9 BAS fun seeking, 10 BAS reward responsiveness, 11 Narcissism: leadership, 12 Narcissism: grandiose, 13 Narcissism: entitlement

| **Variables** | **1** | **2** | **3** | **4** | **5** | **6** | **7** | **8** | **9** | **10** |
| --- | --- | --- | --- | --- | --- | --- | --- | --- | --- | --- |
| Total pay-off | -0.21 | -.37* | -0.23 | -0.2 | -0.12 | 0.11 | -0.19 | 0.28 | 0.29 | .25* |
| Frequency of rule-breaking | -0.2 | -.36* | -0.21 | -0.15 | -0.08 | 0.09 | -0.17 | .27* | .26* | 0.18 |
| Recency | 0.13 | 0 | 0.07 | 0.16 | -0.03 | 0.05 | 0.02 | -0.19 | -0.16 | -0.08 |
| Latency | -0.16 | -.38* | -0.19 | -0.13 | -0.05 | 0.07 | -0.14 | .25* | 0.24 | 0.2 |
| Total time (ms) | -0.08 | 0.08 | .4* | .25* | 0.18 | -0.1 | -0.13 | -.34* | -0.01 | -0.23 |
| Initiation time (ms) | 0.14 | 0.07 | 0.08 | 0.1 | 0.11 | 0.05 | -0.22 | -0.14 | -0.12 | -0.2 |
| Movement time (ms) | -0.18 | 0.02 | .31* | 0.15 | .08* | -0.13 | 0.04 | -0.2 | 0.08 | -0.06 |
| AUC (px^2^) | -0.16 | 0.18 | 0.17 | 0.2 | 0.04 | -0.13 | -0.01 | -0.11 | 0.07 | -0.08 |
| MAD (px) | -0.2 | 0.12 | 0.15 | 0.2 | 0 | -0.09 | -0.03 | -0.09 | 0.08 | -0.04 |

***Table S23.*** Correlations of personality across all variables in rule-breakers when they break the rule

Correlation significant at the .05 level (2 – tailed) represented with asterisk (*).

BIS: Behavioural inhibition system. BAS: Behavioural activation system

1 Risk propensity, 2 Agreeableness, 3 Conscientiousness, 4 Extraversion, 5 Openness, 6 Neuroticism, 7 BIS, 8 BAS drive, 9 BAS fun seeking, 10 BAS reward responsiveness, 11 Narcissism: leadership, 12 Narcissism: grandiose, 13 Narcissism: entitlement
